# Functional Effects of Prebiotic Fructans in Colon Cancer and Calcium Metabolism in Animal Models

**DOI:** 10.1155/2017/9758982

**Published:** 2017-02-15

**Authors:** Marisol Rivera-Huerta, Vania Lorena Lizárraga-Grimes, Ibrahim Guillermo Castro-Torres, Mabel Tinoco-Méndez, Lucía Macías-Rosales, Francisco Sánchez-Bartéz, Graciela Guadalupe Tapia-Pérez, Laura Romero-Romero, María Isabel Gracia-Mora

**Affiliations:** ^1^Unidad de Investigación Preclínica (UNIPREC), Facultad de Química, Universidad Nacional Autónoma de México, Ciudad de México, Mexico; ^2^Colegio de Ciencias y Humanidades, Plantel Sur, Universidad Nacional Autónoma de México, Ciudad de México, Mexico; ^3^Departamento de Genética y Bioestadística, Facultad de Medicina Veterinaria y Zootecnia, Universidad Nacional Autónoma de México, Ciudad de México, Mexico; ^4^Departamento de Patología, Facultad de Medicina Veterinaria y Zootecnia, Universidad Nacional Autónoma de México, Ciudad de México, Mexico

## Abstract

Inulin-type fructans are polymers of fructose molecules and are known for their capacity to enhance absorption of calcium and magnesium, to modulate gut microbiota and energy metabolism, and to improve glycemia. We evaluated and compared the effects of Chicory inulin “Synergy 1®” and inulin from Mexican agave “Metlin®” in two experimental models of colon cancer and bone calcium metabolism in mice and rats. Inulins inhibited the development of dextran sulfate sodium-induced colitis and colon cancer in mice; these fructans reduced the concentration of tumor necrosis factor alpha and prevented the formation of intestinal polyps, villous atrophy, and lymphoid hyperplasia. On the other hand, inulin treatments significantly increased bone densitometry (femur and vertebra) in ovariectomized rats without altering the concentration of many serum biochemical parameters and urinary parameters. Histopathology results were compared between different experimental groups. There were no apparent histological changes in rats treated with inulins and a mixture of inulins-isoflavones. Our results showed that inulin-type fructans have health-promoting properties related to enhanced calcium absorption, potential anticancer properties, and anti-inflammatory effects. The use of inulin as a prebiotic can improve health and prevent development of chronic diseases such as cancer and osteoporosis.

## 1. Introduction

Inulin is a water soluble storage polysaccharide and belongs to a group of nondigestible carbohydrates called fructans, which are a category of nutritional compounds that encompasses naturally occurring plant oligo- and polysaccharides in which one or more fructosyl-fructose linkages comprise the majority of glycosidic bonds [1, 2]. Inulin-type fructans (ITF) are present in significant amounts in several fruits and vegetables [3], represent a group of studied and well-established prebiotics that can escape digestion in the upper gastrointestinal tract, and reach the large intestine virtually intact, where they are quantitatively fermented and act as prebiotic [4]. Inulin stimulates growth of the beneficial flora, namely, bifidobacteria, to a less magnitude lactobacilli and possibly other [5, 6]. Chicory inulin and inulin from Mexican agave were the fructans evaluated in this experimental work. Chicory inulin has been identified as an effective prebiotic to promote active fermentation and lactobacilli proliferation in the large intestine and to enhance calcium digestive absorption and deposition in bones [7]. The Mexican agave plant is an important source of fructans, which had demonstrated bifidogenic effects in animal models [8]; however, ITF of* Agave* genus have been poorly studied for their possible protective effects in others diseases [9], for example, cancer and osteoporosis. Colon cancer is a problem of public health and shows a high mortality rate and may be related to eating habits. In experimental models, administration of ITP reduce growth and metastasising properties of implanted tumor cells in mice; these properties seem to be associated with gut flora-mediated fermentation and production of butyrate [10]. In an experimental model in mice, agave fructans significantly increase the concentration of calcium in plasma and bone and increase osteocalcin concentration. These therapeutical effects prevent bone loss and improve bone formation [11]. The principal aim of this experimental work was to evaluate and to compare the effects of two ITF in experimental models of colon cancer and calcium metabolism in rats and mice.

## 2. Materials and Methods

### 2.1. Inulin-Type Fructans

We used nutritional supplements of inulins. First supplement was inulin from Mexican blue agave (*Agave tequilana* Weber var. azul) “Metlin,” Lot. I04E10, and second supplement was Chicory inulin (*Cichorium intybus*), “Synergy 1,” Lot. IQ1LQ9. An extract of isoflavones, FT108284, was mixed with inulins in the study of calcium experimental model.

### 2.2. Experimental Animals

105 adult male BALB/cAnNhsd mice over 5 weeks of age and weighing 18–22 g and 60 female Hsd:Wistar rats over 8-9 weeks of age and weighing 200 ± 25 g were purchased in Harlan Laboratories of Mexico and were used in all experiments. All animals were housed in plastic cages in a temperature-controlled room with 12 : 12-h light-dark cycles and provided ad libitum access to food and water. Mice and rats had free access to commercial rodent food (Harlan Teklad). Animal care and procedures were conducted according to the guidelines approved by Norma Oficial Mexicana (NOM-062-ZOO-1999) and were subjected to experimental protocols approved by the CICUAL (Institutional Bioethic Committee).

### 2.3. Experimental Model of Colon Cancer

We used the model of azoxymethane and dextran sulfate sodium- (AOM/DSS-) induced colon cancer in mice previously reported [12]. Mice were treated with a single injection of azoxymethane at doses of 10 mg/kg i.p., and subsequently the mice were treated with an aqueous solution of DSS 2% during 4 days. Different experimental groups and treatments are shows in Table 1. After experimental protocol, mice were sacrificed by CO_2_ asphyxiation.

### 2.4. ELISA Analysis for Model Cancer

Colon and jejunum specimens were prepared and washed with Buffer solution (PBS). We analyzed different samples of intestine in order to take advantage of the experimental model and investigate major alterations in the intestinal tract of the mice. Specimens were frozen (−20°C) and stored. Intestine was cut longitudinally or everted and mucosa was scraped and homogenized with 0.5 mL PBS (pH 7.2). The samples were centrifuged at 12000 rpm during 20 minutes. Using the ELISA standardized method was measured the concentration of tumor necrosis factor alpha (TNF-*α*) and interleukin-10 (IL-10) (mouse TNF*α*, BD 5552683017983, and mouse IL-10 BD 5552523217950).

### 2.5. Histopathology Studies for Model of Colon Cancer

Different intestinal specimens of mice were prepared and fixed in 10% neutral buffered formaldehyde embedded in paraffin and 5 *μ*m thin slices were cut and stained with haematoxylin-eosin. All histological sections were reviewed by a pathologist.

### 2.6. Experimental Model of Calcium Metabolism

Loss of ovarian function plays an important role in the development of osteoporosis. Associated with this, bilateral oophorectomy was performed on the 60 rats in the oophorectomized groups and there was a group of 10 rats without oophorectomy. After 15 days after surgery, vaginal secretion was collected with a swab impregnated with distilled water by inserting the tip into the rat vagina, but not deeply. Vaginal fluid was placed on two glass slides. A glass slide was dipped in 70% alcohol for 15 minutes and dried at room temperature and the other was left unfixed in alcohol. The determination of the estrous cycle was studied by Papanicolaou and Diff-Quick staining methods. Different experimental groups and treatments are shows in Table 2. Also we reported the variation of weight in rats during experimental study.

*Biochemical Analysis for Model of Calcium Metabolism*. Three blood analyses were performed: at the beginning of the experiment, after a month, and after two months of treatments. All rats were weighed and anesthetized (isoflurane) and were blood drawn from the retro-orbital vein after an overnight fast. Serum was separated by centrifugation and determination of biochemical parameters was carried out using a photocolorimeter Dayton Randox. Associated with urinalysis, rats were maintained in metabolic cages and a urine sample was collected in the three times above.

### 2.7. Bone Densitometry

At the end of the experimental period, rats were euthanized in CO_2_ chambers. Femur and T13 vertebra were collected and properly stored. the bone samples were analyzed by dual-energy X-ray absorptiometry, in which the bone was exposed to small dose of ionizing radiation to produce pictures of the inside of the rats' body. Bone mineral density (BMD) was given by the bone mineral content, using a densitometer (Discovery QDR series Hologic) with special software for rats.

### 2.8. Statistical Analysis

We applied different statistical analysis according to experimental model used (IBM SPSS 19 Statistics software). We used a generalized linear model for the analysis of concentration of IL-10 and TNF*α* and ANOVA for the analysis of serum biochemical parameters.

## 3. Results 

### 3.1. Model of Colon Cancer

**Figure 1:**
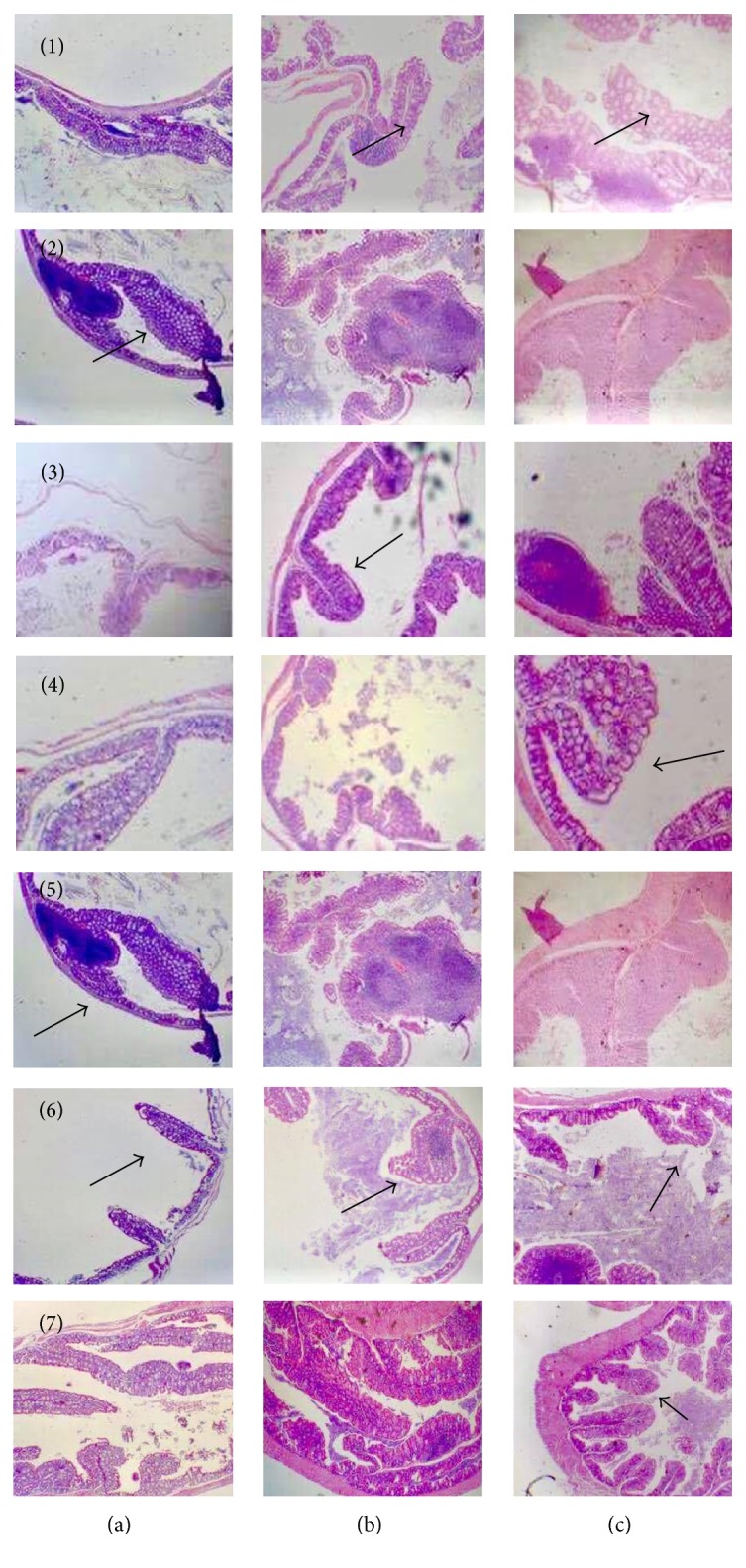
Histopathological studies in intestinal tissues of mice. Inulins treatments diminished the number of intestinal polyps (indicated by black arrows) compared with control group, which showed damage in intestinal mucosa. Samples were analyzed in three different times of treatment: (a) three months, (b) six months, and (c) nine months. (1) Synergy 1-preinduction. (2) Metlin-preinduction. (3) Synergy 1-pre- and postinduction. (4) Metlin- pre- and postinduction. (5) Synergy 1-postinduction. (6) Metlin-postinduction. (7) Control.

The histopathology study of different experimental groups showed changes in intestinal mucosa during the development of cancer. Treatment with ITF diminished intestinal polyps compared with experimental group without fructans treatment. Administration of AOM/DSS caused important changes in intestinal mucosa; for example, there is a thickening of the bowel wall. Results of histopathology are showed in Figure 1.

**Figure 2:**
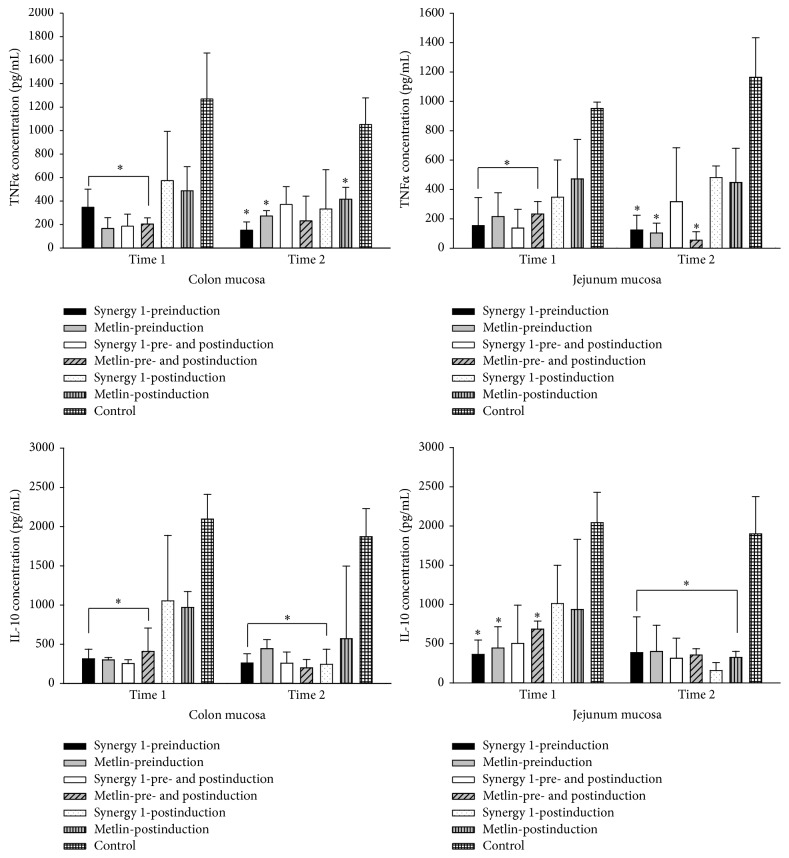
TNF*α* and IL-10 concentration in intestinal samples. Values are presented as means ± SE using 5 data of TNF*α* and IL-10 concentration. ^*∗*^Values indicate significant differences (*p* < 0.05) between groups treated with fructans versus cancer-induced group. Time 1, seven months. Time 2, nine months.

*Concentration of IL-10 and TNFα*. Concentration of TNF*α* and interleukin- (IL-) 10 in the AOM/DSS group was significantly increased in the colon and jejunum (Figure 2). Concentration of TNF*α* was significantly decreased in all groups treated with ITF compared with control group, which development colon cancer. During the different treatments, inulins showed beneficial effects in intestinal mucosa of mice. Results about the concentration of TNF*α* and IL-10 are showed in Figure 2.

### 3.2. Model of Calcium Metabolism

**Figure 3:**
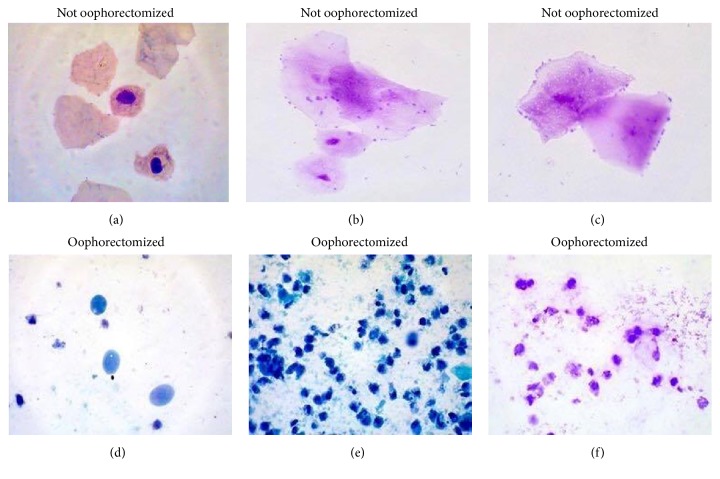
Histology in rats with and without oophorectomy. Vaginal smear from not oophorectomized animal. (a) Anuclear squamae are seen. Papanicolaou stain ×40. (b) and (c) Diff-Quik stain. (d) Parabasal epithelial cells are seen in vaginal smear from an oophorectomized rat, Papanicolaou stain ×40. (e) and (f) Diff-Quik stain.

*Histology and Calcium Concentration*. In rats with oophorectomy, the vaginal smear samples were heavily populated with dark blue leukocytes and nucleated cells with pink or blue cytoplasm. Some cornified cells also were present in the sample. Smears of the not oophorectomized rats revealed the dominance of superficial and anuclear cells and also cornified cells. Histological results after oophorectomy on rats are shown in Figure 3. Histology results in femur of rats showed that different fructan treatments did not produce damage.

A significant weight increase was observed in oophorectomized rats with inulins and isoflavones treatments, compared with control rats not oophorectomized. Calcium serum concentration was compared between different experimental groups. There were no apparent changes referring to calcium concentration after treatment with inulins. Oophorectomized rats showed low concentration of serum calcium; however this result does not have statistical significance compared with other experimental groups. 

*Biochemical Studies.* The results of biochemical analysis are shown in Table 3. Some biochemical parameters are significant between different experimental groups treated with inulins; however, all results indicate that there were no significant changes in the plasma concentration compared with normal biochemical values reported in the bibliography. 

*Bone Densitometry.* Bone densitometry was significantly increased (*p* < 0.05) by the administration of inulin from Mexican agave “Metlin” with isoflavones (0.243 g/cm^2^). All experimental groups treated with ITF increased bone densitometry in femur and vertebra compared with oophorectomized and not oophorectomized rats. The best results about bone mineral density were observed after treatment with inulin of Mexican agave (Metlin) compared with inulin from Chicory. The results are showed in Table 4.

## 4. Discussion

In this experimental work, we evaluated and compared the effects of two ITF in different experimental models of colon cancer and calcium metabolism. ITF can be considered as prebiotics and their consumption has been associated with beneficial health effects, for example, to improve lipid profile, antioxidant function, and colon cancer prevention and to improve bioavailability of calcium and magnesium [13]. In our results about AOM/DSS-induced colon cancer in BALB/cAnNhsd mice model, there were several histopathological changes in intestinal mucosa, such as villous atrophy, polyp formation, intestinal cell dysplasia, and lymphoid hyperplasia. These histopathological effects were similar to results reported by Suzuki and coworkers; however, these authors demonstrated fewer intestinal polyps in mucosa [12, 14]. Another very important aspect is the role played by the intestinal microflora in the development of cancer. The symbiotic interactions between gut microbiota and the digestive tract highly contribute to maintain the gut homeostasis, but the alterations of these interactions caused by environmental changes (infection, diet, and lifestyle) can promote damage in colon mucosa (dysplasia) and development of colorectal cancer [15]. Our experimental work reported dysplasia in intestinal tissue; however, we do not evaluate the possible interactions between different microorganism and their association after ITF treatments. Our two inulins from Mexican blue agave and from Chicory showed beneficial effects in intestine, which prevented development of colorectal cancer. One of the action mechanisms of our two inulins could be associated with the microorganism in gut. Besides the consumption of probiotics to stimulate favorable bacterial communities in the human gastrointestinal tract, prebiotics such as ITF can be consumed to increase the number of bifidobacteria in the colon. Several functions have been attributed to bifidobacteria, encompassing degradation of nondigestible carbohydrates, protection against pathogens, production of vitamin B, antioxidants, and conjugated linoleic acids, and stimulation of the immune system [16]. Our inulins from agave and from Chicory could produce fermentation products such as butyrate and propionate; these molecules inhibit the growth of colon tumor cells and histone deacetylases. Butyrate also causes apoptosis, reduces metastasis in colon cell lines, and protects from genotoxic carcinogens by enhancing expression of enzymes involved in detoxification [17].

Mexican agave plant (*Tequilana *Weber Blue Variety) is an interesting source of fructans, which are formed by a complex mix of fructooligosaccharides with prebiotic actions [18, 19]. Indeed, the bifidogenic and physiologic effects of these fructans in vitro and in animal models had been demonstrated [19, 20]. In our experimental study it was not possible to associate the effect of fructans in colon cancer with the role of microorganisms in the small intestine; however, we studied the possible action mechanism involved in TNF*α* and IL-10 concentration. TNF*α* is a multifunctional cytokine that plays important roles in diverse cellular events such as cell survival, proliferation, differentiation, and death [21]. As a proinflammatory cytokine, TNF is secreted by inflammatory cells, which may be involved in inflammation-associated carcinogenesis [22]. On the other hand, IL-10 is a regulatory cytokine essential to the intestinal immune system. It modulates both the innate and adaptive immune responses, suppresses effector T cell responses, and helps maintain mucosal homeostasis [23]. Our results showed that experimental groups treated with ITF significantly diminished concentration of TNF*α* compared with cancer-induced experimental group, which showed an important increase of TNF*α* concentration. These results are similar to those reported by other authors [24, 25]. The results we obtained on IL-10 were controversial. In our experimental group treated with AOM/DSS there was an increase of IL-10 concentration and this would represent an unfavorable prognostic factor for mice with cancer; however if we consider all the damage to the intestinal mucosa by the administration of DSS, IL-10 can increase its concentration, because it is playing a role of immunological tolerance.

In the other section of our experimental study, we showed that ITP significantly increased bone densitometry in femur and vertebra in oophorectomized rats. In this experimental model, we can study the loss of bone parameters, due to the fact that estrogen deficiency is an important risk factor for the development of bone diseases. Prebiotics, including ITF, have a beneficial effect on calcium bioavailability [5]. Our results demonstrated that calcium concentration in blood was not altered after inulins treatment. Fructans from Mexican agave showed a decrease of calcium concentration in urine; these results can be associated with a favorable calcium absorption in small intestine. Bone densitometry was significantly increased after treatment with Metlin + isoflavones to difference with fructans from Chicory. Fructans from Mexican agave provide several health benefits and have excellent technological properties, but only few data report their physiological effect when added in the diet [26, 27]. The different types of fructans studied in the present experiment seem to have similar activity on mineral absorption. However, the combination of inulin with isoflavones showed synergistic effects on calcium absorption and balance in rats. The mechanism of action in increasing absorption is unknown but may be related to increased colonic calcium bioavailability.

## 5. Conclusions

The data suggest that fructans from Chicory (Synergy 1) and from Mexican agave (Metlin) may contribute to a reduction in the risk of colon cancer. The action mechanism could be associated with a decrease of TNF*α* concentration. The different types of fructans studied in the present experiment seem to have similar activity on calcium deposition in bone. However, inulin from Mexican agave and its combination with isoflavones showed synergistic effects to favor the deposition of calcium. Further studies with other combinations of fructans need to be done to extend these findings.

## Figures and Tables

**Table 1:** Experimental design of colon cancer model.

Number	Experimental group	Treatment
1	Synergy 1-preinduction	1 month with inulin treatment/AOM·DSS/8 months without inulin treatment
2	Metlin-preinduction	

3	Synergy 1-pre- and postinduction	1 month with inulin treatment/AOM·DSS/8 months with inulin treatment
4	Metlin-pre- and postinduction	

5	Synergy 1-postinduction	AOM·DSS/9 months with inulins treatment
6	Metlin-postinduction	

7	Control	Administration of AOM·DSS without inulins treatment

**Table 2:** Experimental design of calcium metabolism.

Number	Experimental group	Treatment
1	Metlin	Inulins were administered at doses of 385 mg/day/rat. Inulins were dissolved in water and the rats had free access to water and normal diet
2	Synergy 1	

3	Isoflavones	Isoflavones were administered at doses of 33.5 mg/day/rat at the same conditions as that of the inulins

4	Metlin + isoflavones	A mixture of inulins and isoflavones was administered under doses indicated above
5	Synergy 1 + isoflavones	

6	Without inulins	Oophorectomized experimental group without inulins treatments

7	Without inulins	Experimental group not oophorectomized without inulins treatment

All the rats of experimental groups 1–6 were oophorectomized and only the experimental group 7 had rats without oophorectomy.

**Table 3:** 

Biochemical parameter	Normal data	Experimental groups						
		1	2	3	4	5	6	7
AST	114.5 ± 25.4	174 ± 5.4^a^	178.5 ± 5.4^a^	183 ± 5.4^a^	182 ± 5.4^a^	181 ± 5.4^a^	166.3 ± 5.4^a^	172 ± 5.4^a^
ALT	58.5 ± 10.2	51.2 ± 1.8^ab^	54.6 ± 1.8^b^	52.7 ± 1.8^ab^	54.3 ± 1.8^b^	53.9 ± 1.8^ab^	47.1 ± 1.8^a^	51 ± 1.8^ab^
FAS	389.7 ± 49.3	349 ± 20.53^a^	350.2 ± 20.53^a^	338.8 ± 20.53^a^	363.4 ± 20.53^a^	379 ± 20.53^a^	359.6 ± 20.53^a^	303.7 ± 20.53^a^
Glucose	7.9 ± 0.8	7.8 ± 0.26^a^	7.7 ± 0.26^a^	7.5 ± 0.26^a^	7.4 ± 0.26^a^	7.3 ± 0.26^a^	6.9 ± 0.26^a^	7.1 ± 0.26^a^
Cholesterol	2.0 ± 0.5	2 ± 0.08^ab^	1.83 ± 0.08^ab^	2 ± 0.08^ab^	2 ± 0.08^ab^	2.2 ± 0.08^b^	1.9 ± 0.08^ab^	1.78 ± 0.08^a^
Triglycerides	0.66 ± 0.16	0.9 ± 0.1^ab^	0.61 ± 0.1^a^	0.72 ± 0.1^ab^	0.62 ± 0.1^ab^	0.75 ± 0.1^ab^	0.82 ± 0.1^ab^	1.15 ± 0.1^b^
Creatinine	50.7 ± 13.0	68.4 ± 2.2^ab^	68.5 ± 2.2^ab^	70.4 ± 2.2^bd^	62 ± 2.2^abc^	70 ± 2.2^bcd^	64.3 ± 2.2^ab^	61.2 ± 2.2^a^
Phosphorus	1.54 ± 0.26	2.16 ± 0.06^a^	2.1 ± 0.06^a^	1.85 ± 0.06^a^	2.06 ± 0.06^a^	2.03 ± 0.06^a^	1.94 ± 0.06^a^	1.98 ± 0.06^a^
Calcium	2.80 ± 0.03	2.66 ± 0.05^b^	2.57 ± 0.05^ab^	2.50 ± 0.05^ab^	2.58 ± 0.05^ab^	2.47 ± 0.05^ab^	2.34 ± 0.05^a^	2.57 ± 0.05^ab^
^*∗*^Creatinine	4,878.80 ± 437.58	4,348 ± 342.13^a^	4,139 ± 342.13^a^	4,157 ± 342.13^a^	3,979 ± 342.13^a^	4,260 ± 342.13^a^	4,069 ± 342.13^a^	3,019 ± 342.13^b^
^*∗*^Calcium	1.43 ± 0.082	1.48 ± 0.19^a^	1.60 ± 0.19^a^	1.80 ± 0.19^a^	1.32 ± 0.19^ac^	1.93 ± 0.19^ab^	1.83 ± 0.19^a^	1.61 ± 0.19^a^

^*∗*^Biochemical parameter in urine. Others parameters were analyzed in blood. AST, ALT, and FAS are expressed in IU/L, and glucose, cholesterol, triglycerides, phosphorus, and calcium are expressed in mmol/L and creatinine in *μ*mol/L. Different letters indicate significant differences (*p* < 0.05) between all experimental groups. Experimental groups, 1, Metlin; 2, Synergy 1; 3, isoflavones; 4, Metlin + isoflavones; 5, Synergy 1 + isoflavones; 6, oophorectomized rats without inulins treatment; 7, not oophorectomized rats without inulins treatment.

**Table 4:** 

Bone	Experimental groups						
	1	2	3	4	5	6	7
Femur	0.231 ± .003^abc^	0.239 ± .003^bdc^	0.234 ± .003^abdc^	0.243 ± .003^bd^	0.236 ± .003^bdc^	0.225 ± .003^ac^	0.233 ± .003^abc^
Vertebra	0.212 ± .004^bd^	0.205 ± .004^abe^	0.207 ± .004^abe^	0.220 ± .004^bdf^	0.195 ± .004^ace^	0.195 ± .004^ae^	0.205 ± .004^abe^

Different letters indicate significant differences (*p* < 0.05) between all experimental groups. Experimental groups, 1, Metlin; 2, Synergy 1; 3, isoflavones; 4, Metlin + isoflavones; 5, Synergy 1 + isoflavones; 6, oophorectomized rats without inulins treatment; 7, not oophorectomized rats without inulins treatment.

## References

[1] Neyrinck A. M., Pachikian B., Taminiau B. (2016). Intestinal sucrase as a novel target contributing to the regulation of glycemia by prebiotics. *PLoS ONE*.

[2] Roberfroid M. B. (2005). Introducing inulin-type fructans. *British Journal of Nutrition*.

[3] Kelly G. (2008). Inulin-type prebiotics—a review: part 1. *Alternative Medicine Review*.

[4] Shoaib M., Shehzad A., Omar M. (2016). Inulin: properties, health benefits and food applications. *Carbohydrate Polymers*.

[5] Krupa-Kozak U., Świątecka D., Bączek N., Brzóska M. M. (2016). Inulin and fructooligosaccharide affect: in vitro calcium uptake and absorption from calcium-enriched gluten-free bread. *Food and Function*.

[6] Roberfroid M. B. (2007). Inulin-type fructans: functional food ingredients. *Journal of Nutrition*.

[7] Demigné C., Jacobs H., Moundras C. (2008). Comparison of native or reformulated chicory fructans, or non-purified chicory, on rat cecal fermentation and mineral metabolism. *European Journal of Nutrition*.

[8] López-Velázquez G., Díaz-García L., Anzo A. (2013). Safety of a dual potential prebiotic system from Mexican agave “Metlin and Metlos”, incorporated to an infant formula for term newborn babies: a randomized controlled trial. *Revista de Investigacion Clinica*.

[9] Dávila-Céspedes A., Juárez-Flores B. I., Pinos-Rodríguez J. M. (2014). Protective effect of *Agave salmiana* fructans in azoxymethane-induced colon cancer in wistar rats. *Natural Product Communications*.

[10] Pool-Zobel B. L. (2005). Inulin-type fructans and reduction in colon cancer risk: review of experimental and human data. *British Journal of Nutrition*.

[11] García-Vieyra M. I., Del Real A., López M. G. (2014). Agave fructans: their effect on mineral absorption and bone mineral content. *Journal of Medicinal Food*.

[12] Suzuki R., Kohno H., Sugie S., Nakagama H., Tanaka T. (2006). Strain differences in the susceptibility to azoxymethane and dextran sodium sulfate-induced colon carcinogenesis in mice. *Carcinogenesis*.

[13] dos Reis S. A., da Conceição L. L., Rosa D. D., Dias M. M. D. S., Peluzio M. D. C. G. (2015). Mechanisms used by inulin-type fructans to improve the lipid profile. *Nutricion Hospitalaria*.

[14] Perše M., Cerar A. (2012). Dextran sodium sulphate colitis mouse model: traps and tricks. *Journal of Biomedicine and Biotechnology*.

[15] Gagnière J., Raisch J., Veziant J. (2016). Gut microbiota imbalance and colorectal cancer. *World Journal of Gastroenterology*.

[16] Rivière A., Selak M., Lantin D., Leroy F., De Vuyst L. (2016). Bifidobacteria and butyrate-producing colon bacteria: importance and strategies for their stimulation in the human gut. *Frontiers in Microbiology*.

[17] Hu S., Dong T. S., Dalal S. R. (2011). The microbe-derived short chain fatty acid butyrate targets miRNA-dependent p21 gene expression in human colon cancer. *PLoS ONE*.

[18] Praznik W., Löppert R., Rubio J. M., Zangger K., Huber A. (2013). Structure of fructo-oligosaccharides from leaves and stem of Agave tequilana Weber, var. azul. *Carbohydrate Research*.

[19] Gomez E., Tuohy K. M., Gibson G. R., Klinder A., Costabile A. (2010). In vitro evaluation of the fermentation properties and potential prebiotic activity of Agave fructans. *Journal of Applied Microbiology*.

[20] Cieslik E., Topolska K., Praznik W., Cruz Rubio J. M. (2012). Effect of Agave fructans on selected parameters of calcium metabolism and bone condition in rats. *The Journal of Aging Research & Clinical Practice*.

[21] Wang X., Lin Y. (2008). Tumor necrosis factor and cancer, buddies or foes?. *Acta Pharmacologica Sinica*.

[22] Wullaert A., Heyninck K., Beyaert R. (2006). Mechanisms of crosstalk between TNF-induced NF-*κ*B and JNK activation in hepatocytes. *Biochemical Pharmacology*.

[23] Wu C., Xu Z., Gai R., Huang K. (2016). Matrine ameliorates spontaneously developed colitis in interleukin-10-deficient mice. *International Immunopharmacology*.

[24] Rodriguez-Canales M., Jimenez-Rivas R., Canales-Martinez M. M. (2016). Protective effect of *Amphipterygium adstringens* extract on dextran sulphate sodium-induced ulcerative colitis in mice. *Mediators of Inflammation*.

[25] Fluckiger A., Dumont A., Derangère V. (2016). Inhibition of colon cancer growth by docosahexaenoic acid involves autocrine production of TNF*α*. *Oncogene*.

[26] Abrams S. A., Hawthorne K. M., Aliu O., Hicks P. D., Chen Z., Griffin I. J. (2007). An inulin-type fructan enhances calcium absorption primarily via an effect on colonic absorption in humans. *Journal of Nutrition*.

[27] Gracia M. I., Tinoco M. M., Rivera H. M. (2013). Acute Toxicity and Genotoxic Evaluation of Metlin and Metlos (Organic Agave Fructans). *Food and Nutrition Sciences*.

